# Comparison of Conventional Therapies With Stem Cell Therapy in the Treatment of Heart Disease

**DOI:** 10.7759/cureus.91077

**Published:** 2025-08-26

**Authors:** Muhammad Rehan Mushtaq, Shafaq Rubab, Ushna Riaz, Sardar Saif Haroon, Shahzaib Ahmed, Francis Asante Baadu

**Affiliations:** 1 Orthopaedics, Luton and Dunstable University Hospital, Luton, GBR; 2 Internal Medicine, Islam Teaching Hospital, Sialkot, PAK; 3 Acute Medicine, Leicester Royal Infirmary, Leicester, GBR; 4 General Medicine, George Eliot Hospital National Health Service (NHS) Trust, Nuneaton, GBR; 5 Cardiology, Nottingham University Hospital National Health Service (NHS) Trust, London, GBR; 6 Internal Medicine, Komfo Anokye Teaching Hospital, Kumasi, GHA

**Keywords:** effective, pateints, quality of life (qol), stem cell, therapies

## Abstract

Background: Heart disease remains a leading cause of mortality and morbidity worldwide. Conventional treatment options, including pharmacological therapies and surgical interventions, have been effective in managing symptoms and preventing further progression of the disease.

Objective: This study aims to compare the efficacy and safety of stem cell therapy with conventional therapies in patients with heart disease.

Methods: This prospective cohort study was conducted at Nottingham University Hospital, NHS, UK, from January 2024 to December 2024. A total of 95 patients were added to the study. Patients were divided into two groups: 47 patients received stem cell therapy, and 48 patients received conventional therapy, which included pharmacological treatments, surgical procedures, or implantable devices. The primary outcomes assessed were improvement in left ventricular ejection fraction (LVEF), exercise capacity measured by the six-minute walk test, and quality of life using the Minnesota Living with Heart Failure Questionnaire (MLHFQ).

Results: The stem cell therapy group showed significant improvements in left ventricular ejection fraction (LVEF), increasing from 30.2% ± 8.4% at baseline to 43.6% ± 9.7% after six months (p < 0.01). In contrast, the conventional therapy group showed a smaller increase in LVEF, from 32.5% ± 7.9% to 36.8% ± 8.1% (p = 0.04). Exercise capacity, measured by the six-minute walk test, improved by 80 meters in the stem cell group compared to a 30-meter improvement in the conventional group (p < 0.001). Quality of life, assessed using the MLHFQ, improved significantly more in the stem cell group, with a reduction in score from 56.2 ± 15.3 to 38.5 ± 12.1 (p < 0.001), versus a reduction from 54.7 ± 14.5 to 48.6 ± 13.2 (p = 0.02) in the conventional therapy group. Hospitalization rates were lower in the stem cell group (10.6%) compared to the conventional group (27.1%) (p = 0.03).

Conclusions: It is concluded that stem cell therapy offers significant advantages over conventional therapies in the management of heart disease, particularly in terms of improving cardiac function, exercise capacity, and quality of life. Unlike previous studies that evaluated stem cell therapy in isolation or without direct comparison, this article uniquely provides a head-to-head analysis against conventional treatments in a prospective cohort, offering practical insights into how stem cell therapy may complement or outperform current standards of care.

## Introduction

Heart disease, particularly ischemic heart disease, heart failure, and myocardial infarction, remains one of the leading causes of morbidity and mortality worldwide, exerting a significant burden on both individuals and healthcare systems [1]. The conventional methods of treatment, such as pharmacological interventions, surgical procedures, and lifestyle changes, have played a critical role in the control of the different heart illnesses [2]. Such therapies are, however, associated with limitations like prolonged dependence on medicines, a great possibility of recurrence, and poor results with specific groups of patients [3]. Consequently, there has been growing interest in exploring novel treatment strategies that can overcome these limitations and offer more sustainable, long-term solutions [4]. Stem cell therapy is one such new therapy that has shed light as a potential treatment for heart disease, especially in the case of a patient with a chronic or terminal form of heart disease, such as ischemic heart disease, heart failure, and myocardial infarction. Regeneration and tissue replacement with stem cells provide a bright hope of reversing heart disease and improving cardiovascular health, with a possibility of restoration of damaged heart tissue by the regeneration and tissue repair process with stem cells [5]. This type of regaining is in contrast to the traditional treatment methods that mainly operated under the control of symptoms and prevention of future harm [6].

Various kinds of stem cells, e.g., embryonic stem cells, induced pluripotent stem cells (iPSCs), and adult stem cells (i.e., mesenchymal stem cells, cardiac progenitor cells, etc.), have been demonstrated in animal experiments and/or human clinical trials [7]. It is thought that these stem cells play their positive role by processes of cell differentiation, out-delivery of paracrine factors, and immune response modulation. Nevertheless, stem cell therapies do not come without complications; moreover, cell sourcing problems, immune rejections, and tumor risks have been experienced [8]. Comparatively, traditional therapeutic approaches, including pharmacologic remedies (e.g., beta-blockers, angiotensin-converting enzyme (ACE) inhibitors), surgery (e.g., coronary artery bypass surgery, cardiac valve repair), and implants (e.g., pacemakers, defibrillators), are still the main artery in the rehabilitation of heart disease [9]. These treatments have been shown to control symptoms and prolong survival, but they do not address the heart muscle's underlying regenerative needs, and their long-term success can be limited by things like disease progression, side effects, and high costs [10]. For instance, pharmacological treatments have been essential in controlling the underlying pathophysiology of heart disease, including reducing blood pressure, controlling cholesterol levels, and improving heart function [11]. It has been demonstrated that angiotensin-converting enzyme (ACE) inhibitors and angiotensin receptor blockers (ARBs) can stop heart failure from getting worse, and beta-blockers can help reduce the workload on the heart and stop arrhythmias. Drugs have been critical in the management of the core pathophysiology of heart disease, including the lowering of blood pressure, the regulation of cholesterol levels, and the enhancement of heart functioning. These four core pillars are renin-angiotensin-aldosterone system (RAAS) blockers, e.g., ACE, ARBs, or ARNIs; evidence-based beta-blockers; mineralocorticoid receptor antagonists (MRAs); and sodium-glucose cotransporter-2 inhibitors (SGLT2). However, these drugs often need to be taken for a long time and may cause side effects like fatigue, drowsiness, and electrolyte imbalances [12]. Additionally, while pharmacological therapies can help manage symptoms, they do not regenerate damaged heart tissue or restore the heart’s functional capacity. Surgical approaches, such as coronary artery bypass grafting (CABG) and percutaneous coronary interventions (PCI), are effective in treating coronary artery disease by restoring blood flow to the heart muscle [13]. However, these procedures do not address the root cause of heart failure or ischemia and often require subsequent interventions or continued medical therapy to prevent recurrence. In addition, surgery may not be an option for all patients, particularly those with advanced heart failure, and its long-term benefits may be limited [14].

This study aims to compare the efficacy and safety of stem cell therapy with conventional therapies in patients with heart disease.

## Materials and methods

This prospective cohort study was conducted at Nottingham University Hospital, NHS, UK, from January 2024 to December 2024. A total of 95 patients were added to the study. Sample size was calculated using the WHO sample size calculator, with a 95% confidence level, 80% power, 5% margin of error, and an expected effect size of 6% improvement in LVEF, resulting in a total of 95 patients. This study was approved by the ethical committee of Nottingham University Hospital, NHS, UK, with IRB number: IRC/1154/23. Inclusion and exclusion criteria of the study are mentioned in Table 1.

**Table 1:** Inclusion and exclusion criteria

Criteria Type	Specific Criteria
Inclusion	Adults aged 18–75 years
Inclusion	Confirmed diagnosis of heart disease (ischemic heart disease, heart failure, or myocardial infarction)
Inclusion	Undergoing conventional therapies or scheduled for stem cell therapy as part of standard care
Exclusion	History of malignancy
Exclusion	Presence of autoimmune diseases
Exclusion	Severe comorbid conditions (e.g., chronic renal failure, advanced pulmonary disease)
Exclusion	Pregnant or breastfeeding women

Data collection

For this study, 95 patients were enrolled consecutively and divided into two groups based on their treatment modality: there were 47 patients where stem cell interventions were done, and 48 patients who were subjected to conventional treatments. Patients of the stem cell therapy group had access to stem cells obtained either by autologous bone marrow or a donor source, according to the clinical conditions and the eligibility of the patient. Standard protocols applied in the administration of the stem cells involved direct myocardial injection or intracoronary infusion. The solution aims to repair damaged heart tissue, thereby enhancing heart performance and eliminating the manifestations of heart disease. The operation was done under local anesthesia, and close observations were carried out to rule out any imminent complications, which may be arrhythmias or any symptoms of immune rejection. The conventional group consisted of the standard treatment of heart disease that could entail drug interventions that consist of beta-blockers, ACE inhibitors, statins, and antiplatelet therapy. Patients might, on some occasions, receive surgical procedures such as coronary artery bypass grafting (CABG) or percutaneous coronary interventions (PCI), depending on the level of their condition. In patients with arrhythmias or high risk of sudden cardiac death, implantable devices, pacemakers/implantable cardioverter-defibrillators (ICDs), can be applied. Primary outcome measures relating to the current study were composed of a number of factors that ensure that these methods of treatment are effective in terms of the treatment modality. Left ventricular ejection fraction (LVEF) (evaluated by means of echocardiography) was used to assess the cardiac work at baseline and following six months of treatment. Quality of life was measured through the Minnesota Living with Heart Failure Questionnaire (MLHFQ), which is a tested and proven instrument that measures the effect of heart failure on the daily life of patients. Patients were observed after six months, with follow-up data being taken at baseline, three, and six months. After every visit, patients are assessed to evaluate the alteration in cardiac functioning, exercise, and quality of life.

Statistical analysis

Data were analyzed using IBM Corp. Released 2020. IBM SPSS Statistics for Windows, Version 26. Armonk, NY: IBM Corp. Descriptive statistics, including means and standard deviations, are used to summarize the baseline characteristics of the study population. The differences between the two treatment groups were analyzed using independent t-tests for continuous variables and chi-square tests for categorical variables. Normality of continuous variables was assessed using the Shapiro-Wilk test prior to applying parametric analyses. The significance level was set at a p-value of 0.05.

## Results

Data were collected from 95 patients. The mean age of patients in the stem cell therapy group was 58.4 ± 10.2 years, while the conventional therapy group had a mean age of 59.1 ± 9.8 years. Gender distribution was comparable, with 59.6% of male patients in the stem cell group (n = 47) and 62.5% in the conventional therapy group (n = 48). Regarding heart disease type, ischemic heart disease was the most common, affecting 48.9% of the stem cell group (n = 23) and 25 (52.1%) of the conventional group. Left ventricular ejection fraction (LVEF) was slightly lower in the stem cell group (30.2% ± 8.4%) compared to the conventional group (32.5% ± 7.9%). The body mass index (BMI) in the stem cell group was 27.4 ± 3.5, while the conventional group had a BMI of 28.1 ± 4.2 (Table 2).

**Table 2:** Demographic and baseline characteristics of the study participants

Characteristic	Stem Cell Therapy (n = 47)	Conventional Therapy (n = 48)	p-value*
Age (years), mean ± SD	58.4 ± 10.2	59.1 ± 9.8	0.72
Gender, n (%)			
Male	28 (59.6%)	30 (62.5%)	0.77
Female	19 (40.4%)	18 (37.5%)	—
Heart Disease Type, n (%)			
Ischemic Heart Disease	23 (48.9%)	25 (52.1%)	0.75
Heart Failure	15 (31.9%)	14 (29.2%)	0.78
Myocardial Infarction	9 (19.1%)	9 (18.8%)	0.96
Left Ventricular Ejection Fraction (LVEF) (%)	30.2 ± 8.4	32.5 ± 7.9	0.19
Body Mass Index (kg/m²), mean ± SD	27.4 ± 3.5	28.1 ± 4.2	0.42
Comorbidities, n (%)			
Hypertension	32 (68.1%)	35 (72.9%)	0.63
Diabetes Mellitus	20 (42.6%)	18 (37.5%)	0.62
Smoking History	14 (29.8%)	12 (25.0%)	0.63

Regarding the left ventricular ejection fraction (LVEF), the stem cell group reported a marked increase in this value (30.2% + 8.4% at baseline to 43.6% + 9.7 at the sixth month) compared to the improvement in the conventional therapy group (32.5% + 7.9 at baseline to 36.8% + 8.1 after six months of treatment) (p < 0.01 and p = 0.04, respectively). The change in the six-minute walk test, the exercise capacity, also favored the stem cell therapy group, although it rose by only 80 meters as compared with the baseline (430.70 meters to 510.65 meters, p < 0.001). The conventional therapy showed less improvement (seventy meters, 420 +/- 60 meters to 450 +/- 75 meters, p = 0.03). The stem cell group experienced an improvement in the quality of life when compared to the conventional therapy group, as evaluated by the Minnesota Living with Heart Failure Questionnaire (MLHFQ), and the decrease in the score was high in the stem cell group, 56.2±15.3 to 38.5±12.1 (p < 0.001), as opposed to the conventional therapy group, 54.7±14.5 to 48.6±13.2 (p = 0.02) (Table 3).

**Table 3:** Improvement in cardiac function (LVEF) in both groups

Group	Baseline LVEF (%)	6-Month LVEF (%)	p-value (LVEF)	t-statistic (LVEF)	χ² (df = 1)	Cohen’s d (LVEF)	Baseline Exercise Capacity (m)	6-Month Exercise Capacity (m)	p-value (Exercise Capacity)
Stem Cell Therapy	30.2 ± 8.4	43.6 ± 9.7	< 0.01	6.02	36.24	1.43	430 ± 70	510 ± 65	< 0.001
Conventional Therapy	32.5 ± 7.9	36.8 ± 8.1	0.04	2.15	4.62	0.51	420 ± 60	450 ± 75	0.03

Only five patients (10.6%) in the stem cell group required hospitalization during the six-month follow-up period, whereas 13 (27.1%) patients in the conventional therapy group were hospitalized (p = 0.03) (Table 4).

**Table 4:** Hospitalization rates in both groups

Group	Hospitalizations (n, %)	p-value	χ² (Chi-square)	df (Degrees of Freedom)	Effect Size (Phi/Cramér’s V)	t-statistic (Hospitalizations)
Stem Cell Therapy	5 (10.6%)	0.03	4.69	1	0.217	-2.11
Conventional Therapy	13 (27.1%)					

In the stem cell therapy group, brain natriuretic peptide (BNP) levels decreased substantially from 852.6 ± 278.5 pg/mL at baseline to 483.1 ± 156.3 pg/mL after six months (p < 0.001), indicating a marked improvement in heart function. In contrast, the conventional therapy group showed a more modest reduction in BNP levels, from 835.4 ± 263.2 pg/mL at baseline to 682.9 ± 190.7 pg/mL (p = 0.04) (Table 5).

**Table 5:** Cardiac biomarkers (BNP) in both groups

Group	Baseline BNP (pg/mL)	6-Month BNP (pg/mL)	p-value (BNP)	t-statistic (BNP)	Effect Size (Cohen’s d, Approx.)
Stem Cell Therapy	852.6 ± 278.5	483.1 ± 156.3	< 0.001	9.82	~1.55 (Large)
Conventional Therapy	835.4 ± 263.2	682.9 ± 190.7	0.04	2.05	~0.35 (Small–Medium)

## Discussion

Their results only indicate that stem cell therapy is of great benefit over the conventional method of treatment in respect of enhancing cardiac health, exercise tolerance, quality of life, and the rate of hospitalization. Although both interventions showed favorable results, they varied greatly with regard to improvement, as the stem cell therapy was significantly higher, showing its importance as a potential new treatment option or addition to the existing means of treating heart disease. This was suggested by the large increase in left ventricular ejection fraction (LVEF) and exercise capacity that was witnessed by the stem cell therapy group because a study has already indicated the possibility of stem cells to regenerate cardiac tissue and enhance heart functioning [15,16]. In our study, a significant increase in the LVEF could be attributed to myocardial repair by stem cell therapy via cellular differentiation and paracrine conveyance. Conversely, ordinary treatment, which works well in alleviating the symptoms, lacks in treating tissue destruction in heart disease. Thus, the effects on LVEF and exercise capacity were also less profound in the standard treatment group.

The six-minute walk test further demonstrated the superior impact of stem cell therapy on exercise capacity. Patients in the stem cell group showed a marked improvement in functional capacity and endurance, reflecting a clinically meaningful reduction in the activity limitations commonly experienced by heart disease patients, such as exertional fatigue and shortness of breath. Although conventional therapies had also shown improvement results, those were lower, indicating that stem cell therapy is more effective and long-lasting. The Minnesota Living with Heart Failure Questionnaire (MLHFQ) measurement of quality of life indicated improved symptom load burden, to a higher degree in the stem cell therapy group. Although both interventions yielded improvements, the stem cell group showed significantly greater gains, which also highlights the potential of stem cell therapy to not only improve the overall well-being of heart disease patients but also to extend life expectancy [17]. This is particularly important in heart failure patients, where symptom burden can significantly affect mental health and social functioning [18]. Although pharmacological and surgical interventions are beneficial, they may not have the same impact on improving the holistic aspects of patients' lives, such as emotional well-being and physical limitations, as evidenced by the modest improvement in the conventional therapy group. The reduction in hospitalizations for heart failure and myocardial infarction observed in the stem cell therapy group is a promising outcome, suggesting that stem cell therapy may help reduce the frequency of acute exacerbations and complications associated with heart disease. This could be attributed to the regenerative effects of stem cells on heart tissue, which may reduce the likelihood of further damage or decompensation. Conventional therapies, though effective in managing symptoms and preventing further deterioration, do not address the underlying heart muscle damage, leading to a higher hospitalization rate in this group. The adverse events in the study were minimal in both groups, with the majority being mild and transient. The occurrence of mild arrhythmias in the stem cell therapy group is consistent with previous studies, which have shown that while stem cell infusion is generally safe, transient arrhythmias may occur due to the myocardial injection process [19]. During the six-month follow-up, adverse events were generally mild and transient in both groups. In the stem cell therapy group, three patients (6.4%) experienced short-lived arrhythmias following intracoronary infusion, which resolved spontaneously without long-term complications. In the conventional therapy group, four patients (8.3%) developed procedure-related complications, most commonly minor infections following coronary artery bypass grafting. Despite the promising results, this study has some limitations. First, the sample size of 95 patients may be relatively small, and larger, multi-center trials would be needed to confirm these findings and determine the generalizability of the results. Additionally, the non-randomized allocation of patients to the treatment groups may introduce selection bias, as patients were not randomly assigned to receive either stem cell therapy or conventional therapy. Although the baseline characteristics of the two groups were comparable, unmeasured confounders could still affect the outcomes.

## Conclusions

It is concluded that stem cell therapy offers significant advantages over conventional therapies in improving cardiac function, exercise capacity, and quality of life in patients with heart disease. The findings of this study demonstrate that stem cell therapy not only leads to more substantial improvements in left ventricular ejection fraction (LVEF) but also enhances physical endurance and reduces the burden of heart failure symptoms. The greater reduction in hospitalization rates and improvements in cardiac biomarkers, such as brain natriuretic peptide (BNP), further suggest that stem cell therapy provides a more robust and lasting therapeutic effect compared to traditional pharmacological and surgical interventions.
